# Characterization of Major and Clinically Relevant Non-Major Bleeds in the APEX Trial

**DOI:** 10.1055/s-0039-1685496

**Published:** 2019-04-17

**Authors:** Megan K. Yee, C. Michael Gibson, Tarek Nafee, Mathieu Kerneis, Yazan Daaboul, Serge Korjian, Gerald Chi, Fahad AlKhalfan, Adrian F. Hernandez, Russell D. Hull, Alexander T. Cohen, Samuel Z. Goldhaber

**Affiliations:** 1 Boston Clinical Research Institute, Newton, Massachusetts, United States; 2 PERFUSE Study Group, Division of Cardiovascular, Departments of Medicine, Beth Israel Deaconess Medical Center, Harvard Medical School, Boston, Massachusetts, United States; 3 Duke University and Duke Clinical Research Institute, Durham, North Carolina, United States; 4 Division of Cardiology, R.A.H. Faculty of Medicine, University of Calgary, Calgary, Alberta, Canada; 5 Department of Haematological Medicine, Guy's and St Thomas' Hospitals, London, United Kingdom; 6 Division of Cardiovascular, Brigham and Women's Hospital, Harvard Medical School, Boston, Massachusetts, United States

**Keywords:** venous thromboembolism, betrixaban, acutely ill patients, major bleed, clinically relevant non-major bleeding

## Abstract

**Background** Among medically ill patients treated with thromboprophylaxis, betrixaban was not associated with an increase in major bleeding compared with enoxaparin, but an increase in clinically relevant non-major (CRNM) bleeding was observed. The aim of this analysis is to describe the severity and clinical consequences of major and CRNM bleeding in the APEX trial.

**Methods** The APEX trial randomized 7,513 hospitalized acutely ill medical patients to receive either enoxaparin for 6 to 14 days or betrixaban for 35 to 42 days. Subjects receiving a concomitant strong p-glycoprotein inhibitor or with creatinine clearance <30 mL/min were administered a reduced dose of study drug.

**Results** A total of 25 (0.7%) and 21 (0.6%) major bleeds occurred in the betrixaban and enoxaparin arms, respectively ( *p* = NS) and a total of 91 (2.5%) and 38 (1.0%) CRNM bleeds occurred in the betrixaban and enoxaparin arm ( *p* < 0.001), respectively. Most major bleeds were considered moderate or severe and most CRNM bleeds were considered mild and moderate ( *p* = NS). One fatal major bleed occurred in each treatment arm. Rates of major or CRNM bleeds resulting in new or prolonged hospitalization (major: 44.0 vs. 28.6%; CRNM: 12.1 vs. 21.1%) or study treatment interruption or cessation (major: 72.0 vs. 71.4%; CRNM: 71.3 vs. 68.4%) were similar between treatment arms ( *p* = NS).

**Conclusions** In the APEX trial, CRNM bleeds were mild or moderate in nature and had less of a clinical impact than major bleeds. The severity and clinical sequela of bleeds in the betrixaban arm were comparable to those in the enoxaparin arm.

**Clinical Trial Registration** URL: http://www.clinicaltrials.gov .; Unique identifier: NCT01583218.

## Introduction

Acutely ill hospitalized patients are at an increased risk for venous thromboembolism (VTE) for at least a month after hospital discharge. 1 2 However, current guidelines recommend the use of parenteral anticoagulants for 6 to 14 days, but do not recommend the use of extended-duration thromboprophylaxis. 3 Trials of extended-duration thromboprophylaxis have demonstrated either a lack of efficacy or efficacy that has been accompanied by an increase in major bleeding. 4 5 6 7 The APEX trial (Acute Medically Ill VTE Prevention with Extended Duration Betrixaban) demonstrated a significant reduction in a composite of asymptomatic deep vein thrombosis (DVT), symptomatic DVT, nonfatal pulmonary embolism (PE), and VTE-related death with extended duration betrixaban compared with standard duration enoxaparin. In contrast to prior studies, betrixaban was not associated with an increase in major bleeds, but was associated with an increase in clinically relevant non-major (CRNM) bleeds. 8 The aim of this post hoc analysis is to describe the severity and clinical impact of major and CRNM bleeds observed in the APEX trial.

## Methods

### Study Population and Design

The APEX trial was a randomized, multicenter, double-blind, double-dummy, placebo-controlled, phase 3 clinical trial. The design and primary results have been previously published. 8 9 Briefly, acutely ill medical patients aged 40 years or older with reduced mobility hospitalized within the last 96 hours for heart failure, respiratory failure, infectious disease, rheumatic disease, or ischemic stroke were eligible for inclusion. Major exclusion criteria included active bleeding, severe renal insufficiency defined as creatinine clearance (CrCl) less than 15 mL/min, or any condition requiring long-term anticoagulation or antiplatelet therapy. Enrolled subjects were randomized in a 1:1 ratio to receive either (1) standard duration active 40 mg enoxaparin once daily for 6 to 14 days plus extended duration oral placebo betrixaban for 35 to 42 days or (2) standard duration enoxaparin placebo for 6 to 14 days plus oral active 80 mg betrixaban for 25 to 42 days. Subjects receiving a concomitant strong P-glycoprotein (P-gp) inhibitor or with renal insufficiency (CrCl < 30 mL/min) received a reduced dose of study drug (40 mg).

### Bleeding Definitions in the APEX Trial

In the main APEX trial, the primary safety endpoint was major bleeding and the secondary safety endpoint was the composite or major or clinically relevant non-major bleeding that occurred within 7 days of all study drug discontinuation. The current analysis will examine major bleeding and CRNM bleeding independently. Major bleeding was defined according to the International Society of Thrombosis and Haemostasis (ISTH) criteria, which includes a clinically overt bleeding that is fatal or associated with a reduction in hemoglobin of at least 2 g/dL, a transfusion of at least 2 units of blood or packed cells, or occurs in a critical area or organ (intraocular, intracranial, intraspinal, intramuscular with compartment syndrome, retroperitoneal bleeding, intraocular bleeding, or pericardial bleeding). 10 CRNM bleeding was defined as an overt bleeding that did not meet the criteria for major bleeding, but was associated with a medical intervention, unscheduled contact with a physician (in person or by telephone), interruption or cessation of study treatment, or discomfort for the subject including pain or impairment of activities of daily living.

### Bleeding Severity

Severity of events was investigator determined. Mild events were defined as events when the subject was aware of signs and symptoms, but the signs and symptoms were easily tolerated. Moderate events were defined as events that caused enough discomfort to interfere with activities of daily living. Severe events were defined as events that caused subjects to be unable to perform normal daily activities. Life-threatening events were those that posed an immediate risk of death.

### Bleeding-Related Clinical Outcomes

Each bleed was assessed to determine if it resulted in any of the following four clinical outcomes: (1) death, (2) requiring or prolonging hospitalization, (3) requiring medical treatment, (4) causing study drug interruption, or (5) study drug cessation.

### Statistical Analysis

Analyses were completed according to the study drug received and in the safety population which includes all subjects who received at least one dose of study drug. Major or CRNM bleeding was tallied in each treatment arm. If a subject experienced more than one bleed, the most severe bleed was counted. Descriptive analyses regarding the location, severity, and clinical outcomes of major and CRNM bleeds were completed according to study drug and dose. The chi-squared test of independence or Fisher's exact test, if any expected cell count was less than 5, was used to test differences between treatment arms.

## Results

### Study Population

A total of 7,513 subjects were randomized in the APEX trial, with 3,759 subjects in the betrixaban arm and 3,754 subjects in the enoxaparin arm. Baseline characteristics were well balanced as previously reported. 8 The safety population was composed of 7,432 subjects who received at least one dose of study drug, with 3,716 subjects in the betrixaban arm and 3,716 in the enoxaparin arm.

### Rates of Major and CRNM Bleeds

As previously reported, the occurrence of major bleeding was similar between the betrixaban arm and the enoxaparin arm (betrixaban = 25 [0.7%], enoxaparin = 21 [0.6%], *p* = 0.55). Rates of major bleeding were unchanged among subjects who received the reduced dose (betrixaban = 10 [1.4%], enoxaparin = 5 [0.7%], *p* = 0.20) and among subjects who received the full dose (betrixaban = 15 [0.5%], enoxaparin = 16 [0.5%], *p* = 0.86). No subjects experienced more than one major bleed. An increase in CRNM bleeding was observed in the betrixaban arm with a total of 91 (2.5%) bleeds compared with 38 (1.0%) bleeds in the enoxaparin arm ( *p* < 0.001). Betrixaban was also associated with an increase in CRNM bleeding compared with enoxaparin among subjects who receive the reduced dose (betrixaban = 25 [3.4%], enoxaparin = 5 [0.7%], *p* < 0.001) and the full dose (betrixaban = 66 [2.2%], enoxaparin = 33 [1.1%], *p* < 0.001). A total of three subjects in the betrixaban arm and one subject in the enoxaparin arm experienced more than one CRNM bleed. No subjects in either arm had both a major bleed and a CRNM bleed.

### Location of Bleeds

**Fig. 1:**
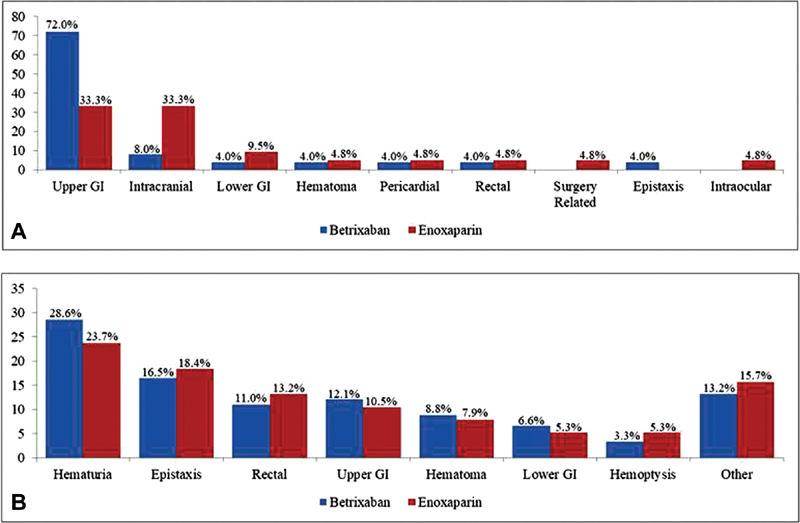
Location of major bleeds ( **A** ) and location of CRNM bleeds ( **B** ). Other bleeds include laceration, hemothorax, vaginal (increased or prolonged menstrual or abnormal vaginal bleeding), bleeding associated with noncardiac surgery, gingival, puncture site, retroperitoneal, subconjunctival/conjunctival, petechial rash/hemorrhage, baker's cyst with intra cyst hemorrhage, and prevertebral space posterior hypopharynx hematoma.

The most common location of major bleeds was upper gastrointestinal (GI), which accounted for 72.0% ( *n* = 18/25) of the major bleeds in the betrixaban arm and 33.3% ( *n* = 7/21) in the enoxaparin arm ( *p* = 0.017). The second most common location for major bleeding was intracranial bleeding which accounted for 8.0% ( *n* = 2/25) in the betrixaban and 33.3% ( *n* = 7/21) in the enoxaparin arm ( *p* = 0.059). Other locations which generally occurred in one patient per treatment arm included lower GI bleeding, hematomas, pericardial bleeding, rectal bleeding, bleeding associated with noncardiac surgery, epistaxis, and intraocular bleeding ( Fig. 1A ; Supplementary Table S1 ).

Locations of CRNM bleeds were comparable between treatment arms ( *p* > 0.05). The most common bleed was gross hematuria accounting for 28.6% ( *n* = 26/91) of CRNM bleeds in the betrixaban arm and 23.7% ( *n* = 9/38) in the enoxaparin arm. Epistaxis accounted for 16.5% ( *n* = 15/91) and 18.4% ( *n* = 7/38) CRNM bleeds in the betrixaban and enoxaparin arm, respectively. Other common locations included rectal bleeding (betrixaban = 11.0% [ *n* = 10/91], enoxaparin = 13.2% [ *n* = 5/38]), upper GI bleeding (betrixaban = 12.1% [ *n* = 11/91], enoxaparin = 10.5% [ *n* = 4/38]), and hematomas (betrixaban = 8.8% [ *n* = 8/91], enoxaparin = 7.9% [ *n* = 3/38]). All CRNM bleed locations are displayed in Fig. 1B and Supplementary Table S2 .

### Severity of Bleeds

**Fig. 2:**
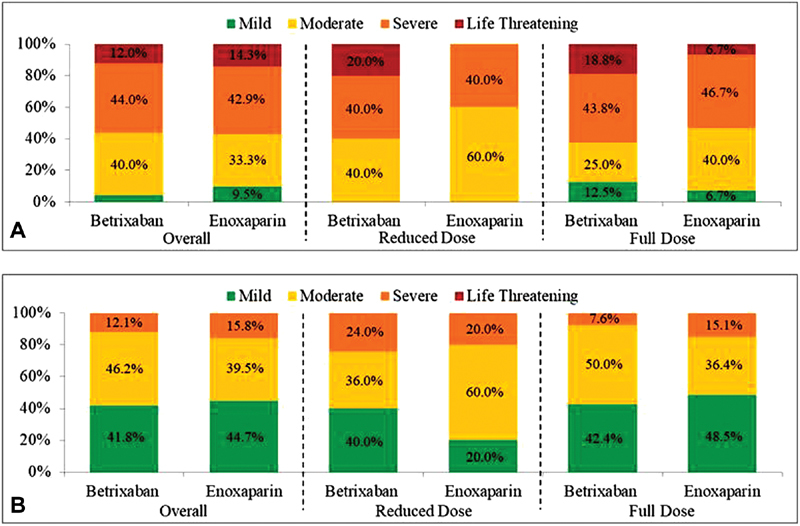
Severity of major bleeds ( **A** ) and severity of CRNM bleeds ( **B** ).

Severity of major bleeding was similar between treatment arms ( *p* > 0.05). Overall, 4.0% ( *n* = 1/25) of major bleeds in the betrixaban arm and 9.5% ( *n* = 2/21) of major bleeds in the enoxaparin arm were considered mild. Most major bleeds were considered moderate with 40.0% ( *n* = 10/25) and 33.3% ( *n* = 7/21) in the betrixaban and enoxaparin arm, respectively, or severe with 44.0% ( *n* = 11/25) in the betrixaban arm and 42.9% ( *n* = 9/21) in the enoxaparin arm. Major bleeds considered life threatening accounted for 12.0% ( *n* = 3/25) in the betrixaban arm and 14.3% ( *n* = 3/21) in the enoxaparin arm. Results were unchanged among subjects receiving the reduced dose and the full dose with the majority of major bleeds considered moderate or severe ( Fig. 2A ; Supplementary Table S3 ).

Severity of CRNM bleeds was also similar between the betrixaban and enoxaparin treatment arms ( *p* > 0.05). Overall, the majority of CRNM bleeds were considered mild (betrixaban = 41.8% [ *n* = 38/91], enoxaparin = 44.7% [ *n* = 17/38]) or moderate (betrixaban = 46.2% [ *n* = 42/91], enoxaparin = 39.5%, [ *n* = 15/38]). A low proportion of CRNM bleeds was considered severe with 12.1% ( *n* = 11/91) and 15.8% ( *n* = 6/38) in the betrixaban and enoxaparin arm, respectively, and no CRNM bleeds were considered life threatening. Results were consistent among subjects receiving the reduced dose and the full dose, with the majority of CRNM bleeds considered mild or moderate, a low proportion considered severe, and no bleeds considered life threatening ( Fig. 2B ; Supplementary Table S4 ).

### Clinical Outcomes of Bleeds

**Fig. 3:**
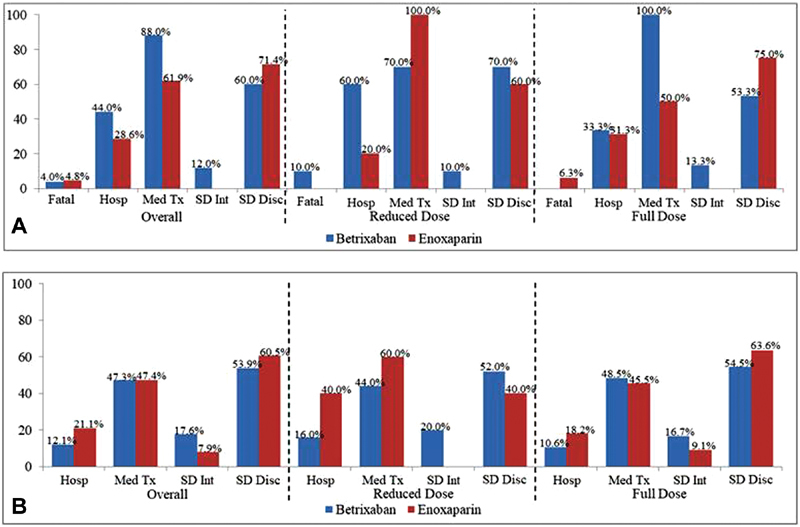
Clinical outcomes of major bleeds ( **A** ) and clinical outcomes of CRNM bleeds ( **B** ). Hosp, required or prolonged hospitalization; Med Tx, required medical treatment; SD Int, study drug interruption; SD Disc, study drug cessation.

One fatal bleed occurred in the betrixaban arm (4.0%) and in the enoxaparin arm (4.8%; *p* > 0.05). Approximately 44.0% ( *n* = 11/25) and 28.6% ( *n* = 6/21) of major bleeds resulted in a new or prolonged hospitalization ( *p* > 0.05). Eighty-eight percent ( *n* = 22/25) of major bleeds in the betrixaban arm and 61.9% ( *n* = 13/21) in the enoxaparin arm required medical treatment ( *p* = 0.019). The majority of major bleeds resulted in study drug cessation (betrixaban = 60.0% [ *n* = 15/25], enoxaparin = 71.4% [ *n* = 15/21]) and a few major bleeds resulted in study drug interruption (betrixaban = 12.0% [ *n* = 3/25]). Results were unchanged among subjects receiving the reduced dose and the full dose ( Fig. 3A ; Supplementary Table S5 ).

No CRNM bleeds resulted in death in either treatment arm. Approximately 12.1% ( *n* = 11/91) of CRNM bleeds in the betrixaban arm and 21.1% ( *n* = 8/38) of CRNM bleeds in the enoxaparin arm resulted in a new or prolonged hospitalization ( *p* > 0.05). CRNM bleeds required medical treatment in 47.3% ( *n* = 43/91) of the betrixaban arm and 47.4% ( *n* = 18/38) of the enoxaparin arm ( *p* < 0.05). Approximately half of CRNM bleeds resulted in study drug cessation in both treatment arms (betrixaban = 53.9% [ *n* = 49/91], enoxaparin = 60.5% [ *n* = 23/38]). Rate of study drug interruption was comparable between treatment arms (betrixaban = 17.6% [ *n* = 16/91], enoxaparin = 7.9% [ *n* = 3/38]). Results remained the same among subjects receiving the reduced dose and the full dose ( Fig. 3B ; Supplementary Table S6 ).

## Discussion

Bleeds experienced in the betrixaban arm were not more severe and did not result in worse clinical outcomes compared with enoxaparin, despite a longer duration of administration of betrixaban. Betrixaban was associated with more GI bleeds and enoxaparin tended to be associated with more intracranial bleeds. This trend of an increase in GI bleeds coupled with a decrease in intracranial bleeds has been previously observed with factor Xa inhibitors. 11 12 13 14

Mitigating intracranial bleeding risk within the context of anticoagulation is of utmost importance given the incidence of anticoagulant-associated intracranial hemorrhages quadrupled over a single decade. 15 Intracranial bleeds are a particular concern not only because of the associated high mortality rate, but also due to the associated burden on healthcare. Intracranial hemorrhage mortality rates are estimated to range between 50 and 57%, 16 17 though only two fatal bleeds occurred throughout the APEX trial duration, one in each treatment arm. Mean hospitalization costs per patient associated with mortality due to an intracranial bleed are estimated to be approximately $76,000. Mean hospitalization costs associated with intracranial bleed survival are estimated even higher at approximately $119,000. 18 In total, the lifetime cost within the U.S. healthcare system is approximately $6.0 billion dollars. 19

Severity patterns and clinical impacts of major and CRNM bleeds were unsurprising. Based on bleeding definitions alone, it is not unexpected that CRNM bleeds were milder and had less of a clinical impact compared with major bleeding. The APEX trial employed an especially sensitive definition for CRNM bleeding which included bleeds that could be reported via telephone without a face-to-face evaluation, and were associated with a hemoglobin drop of less than 2 g/dL. Since the development of the APEX trial, ISTH created a standardized definition for CRNM bleeding, which is more stringent than the APEX criteria, requiring a face-to-face evaluation. 20 In contrast to CRNM bleeding, major bleeding in the APEX trial was defined according to the ISTH definition, which requires the bleed to be fatal, in a critical organ site, or be related with a hemoglobin drop of ≥2 g/dL. 10 Interestingly, the ISTH criteria for major bleeding is also more sensitive than other common standardized major bleeding definitions such as thrombolysis in myocardial infarction (TIMI) major bleeding, which requires bleeds to be fatal, in a critical organ site, or associated with a hemoglobin drop of ≥5 g/dL. 21

Weighing the benefit–risk of a potential bleeding event versus a potential thrombotic event is important when choosing an appropriate anticoagulant treatment and regimen. Given the increased risk of bleeding associated with extended-duration anticoagulation for the prevention of VTE in other studies, potential bleeding concerns remain a critical issue. Betrixaban did not increase major bleeds relative to enoxaparin, but it is still important to consider the increased risk in CRNM bleeding and its meaning to patients. It has previously been demonstrated that both physicians and patients considered death or a disabling stroke as the most important events to avoid, and placed little importance on the prevention of nonfatal major bleeding not requiring a transfusion, moderate bleeding, or CRNM bleeding. 22 Therefore, despite an increase in CRNM bleed, physicians and patients may determine the prophylactic benefits of extended duration betrixaban may outweigh the risk of CRNM bleeding, especially considering the mild to moderate nature of the bleeds observed in the trial.

The current post hoc analyses were not prespecified and the total number of bleeding events was low. Therefore, the results should be interpreted with caution. Additionally, the severity and clinical outcomes were not adjudicated by a clinical events committee. Instead, all outcomes were determined by the site study investigators using their best clinical judgement. However, this form of evaluation best reflects the experience and assessment of these outcomes by physicians and patients in the real world.

## Conclusions

Extended-duration betrixaban was associated with a twofold increase in CRNM bleeding compared with standard duration enoxaparin. The clinical consequences of bleeding were similar between treatment arms without an increase in prolonged or new hospitalizations or medical interventions. Finally, severity of bleeds was consistent between the enoxaparin and betrixaban arms, with most CRNM bleeds considered mild or moderate.
