# Supplementary material for: Characterization of Major and Clinically Relevant Non-Major Bleeds in the APEX Trial
Source: TH Open. 2019 Apr 17;3(2):e103–8. doi: 10.1055/s-0039-1685496 (PMC6524922; doi:10.1055/s-0039-1685496)
Supplement: Supplementary file 1 — Supplementary Material [file 10-1055-s-0039-1685496-s190012.pdf]

Supplementary Table S1 Major bleed locations by treatment arm

	Betrixaban ($N = 25$)	Enoxaparin ($N = 21$)
Upper gastrointestinal	18 (72.0%)	7 (33.3%)
Intracranial	2 (8.0%)	7 (33.3%)
Lower gastrointestinal	1 (4.0%)	2 (9.5%)
Hematoma	1 (4.0%)	1 (4.8%)
Pericardial	1 (4.0%)	1 (4.8%)
Rectal	1 (4.0%)	1 (4.8%)
Bleeding associated with noncardiac surgery	0	1 (4.8%)
Epistaxis	1 (4.0%)	0
Intraocular	0	1 (4.8%)

Notes: Gastrointestinal bleeds $p = 0.009$, intracranial $p = 0.059$, lower gastrointestinal $p = 0.585$, p-value for all other bleeding locations was >0.999.

Supplementary Table S2 Clinically relevant non-major bleeds by treatment arm

	Betrixaban ($N = 91$)	Enoxaparin ($N = 38$)
Hematuria	26 (28.6%)	9 (23.7%)
Epistaxis	15 (16.5%)	7 (18.4%)
Rectal	10 (11.0%)	5 (13.2%)
Upper gastrointestinal	11 (12.1%)	4 (10.5%)
Hematoma	8 (8.8%)	3 (7.9%)
Lower gastrointestinal	6 (6.6%)	2 (5.3%)
Other	5 (5.5%)	2 (5.3%)
Hemoptysis	3 (3.3%)	2 (5.3%)
Laceration	2 (2.2%)	1 (2.6%)
Hemothorax	1 (1.1%)	1 (2.6%)
Vaginal (increased of prolonged menstrual or abnormal bleeding)	2 (2.2%)	0
Bleeding associated with noncardiac surgery	1 (1.1%)	0
Gingival	0	1 (2.6%)
Puncture site	0	1 (2.6%)
Retroperitoneal	1 (1.1%)	0

Supplementary Table S3 Severity of major bleeds

	Betrixaban	Enoxaparin
**Overall**	**$N = 25$**	**$N = 21$**
Mild	1 (4.0%)	2 (9.5%)
Moderate	10 (40.0%)	7 (33.3%)
Severe	11 (44.0%)	9 (42.9%)
Life threatening	3 (12.0%)	3 (14.3%)
**Reduced dose**	**$N = 10$**	**$N = 5$**
Mild	0	0
Moderate	4 (40.0%)	3 (60.0%)
Severe	4 (40.0%)	2 (40.0%)
Life threatening	2 (20.0%)	0
**Full dose**	**$N = 16$**	**$N = 15$**
Mild	2 (12.5%)	1 (6.7%)
Moderate	4 (25.0%)	6 (40.0%)
Severe	7 (43.8%)	7 (46.7%)
Life threatening	3 (18.8%)	1 (6.7%)

Supplementary Table S4 Severity of CRNM bleeds

	Betrixaban	Enoxaparin
**Overall**	**$N = 91$**	**$N = 38$**
Mild	38 (41.8%)	17 (44.7%)
Moderate	42 (46.2%)	15 (39.5%)
Severe	11 (12.1%)	6 (15.8%)
Life threatening	0	0
**Reduced dose**	**$N = 25$**	**$N = 5$**
Mild	10 (40.0%)	1 (20.0%)
Moderate	9 (36.0%)	3 (60.0%)
Severe	6 (24.0%)	1 (20.0%)
Life threatening	0	0
**Full dose**	**$N = 66$**	**$N = 33$**
Mild	28 (42.4%)	16 (48.5%)
Moderate	33 (50.0%)	12 (36.4%)
Severe	5 (7.6%)	5 (15.1%)
Life threatening	0	0

Abbreviation: CRNM, clinically relevant non-major.

Supplementary Table S5 Clinical outcomes of major bleeds

	Betrixaban	Enoxaparin
**Overall**	***N*** **= 25**	***N*** **= 21**
Fatal	1 (4.0%)	1 (4.8%)
Requiring/Prolong hospitalization	11 (44.0%)	6 (28.6%)
Requiring medical treatment[a]	22 (88.0%)	13 (61.9%)
Study drug interruption	3 (12.0%)	0
Study drug cessation	15 (60.0%)	15 (71.4%)
**Reduced dose**	***N*** **= 10**	***N*** **= 5**
Fatal	1 (10.0%)	0
Requiring/Prolong hospitalization	6 (60.0%)	1 (20.0%)
Requiring medical treatment[a]	7 (70.0%)	5 (100.0%)
Study drug interruption	1 (10.0%)	0
Study drug cessation	7 (70.0%)	3 (60.0%)
**Full dose**	***N*** **= 15**	***N*** **= 16**
Fatal	0	1 (6.3%)
Requiring/Prolong hospitalization	5 (33.3%)	5 (31.3%)
Requiring medical treatment[a]	15 (100.0%)	8 (50.0%)
Study drug interruption	2 (13.3%)	0
Study drug cessation	8 (53.3%)	12 (75.0%)

[a]Medical treatment included concomitant medications and nondrug therapies. Overall $p = 0.019$ and full dose $p = 0.002$.

Supplementary Table S6 Clinical outcomes of CRNM bleeds

	Betrixaban	Enoxaparin
**Overall**	***N*** **= 91**	***N*** **= 38**
Fatal	0	0
Requiring/Prolong hospitalization	11 (12.1%)	8 (21.1%)
Requiring medical treatment[a]	43 (47.3%)	18 (47.4%)
Study drug interruption	16 (17.6%)	3 (7.9%)
Study drug cessation	49 (53.9%)	23 (60.5%)
**Reduced dose**	***N*** **= 25**	***N*** **= 5**
Fatal	0	0
Requiring/Prolong hospitalization	4 (16.0%)	2 (40.0%)
Requiring medical treatment[a]	11 (44.0%)	3 (60.0%)
Study drug interruption	5 (20.0%)	0
Study drug cessation	13 (52.0%)	2 (40.0%)
**Full dose**	***N*** **= 66**	***N*** **= 33**
Fatal	0	0
Requiring/Prolong hospitalization	7 (10.6%)	6 (18.2%)
Requiring medical treatment[a]	32 (48.5%)	15 (45.5%)
Study drug interruption	11 (16.7%)	3 (9.1%)
Study drug cessation	36 (54.5%)	21 (63.6%)

Abbreviation: CRNM, clinically relevant non-major.
[a]Medical treatment included concomitant medications and nondrug therapies.
